# Resistant Maltodextrin Intake Reduces Virulent Metabolites in the Gut Environment: A Randomized Control Study in a Japanese Cohort

**DOI:** 10.3389/fmicb.2022.644146

**Published:** 2022-05-04

**Authors:** Yuichiro Nishimoto, Yoshinori Mizuguchi, Yuka Mori, Masaki Ito, Shoko Miyazato, Yuka Kishimoto, Takuji Yamada, Shinji Fukuda

**Affiliations:** ^1^Metagen Inc., Tsuruoka, Japan; ^2^Matsutani Chemical Industry Co., Ltd., Itami, Japan; ^3^Department of Life Science and Technology, Tokyo Institute of Technology, Tokyo, Japan; ^4^Institute for Advanced Biosciences, Keio University, Tsuruoka, Japan; ^5^Intestinal Microbiota Project, Kanagawa Institute of Industrial Science and Technology, Kawasaki, Japan; ^6^Transborder Medical Research Center, University of Tsukuba, Tsukuba, Japan

**Keywords:** prebiotics, gut microbiota, intestinal metabolites, dietary fiber, resistant maltodextrin

## Abstract

In recent years, there have been many reports on the effects of prebiotics on intestinal health. In particular, the consumption of resistant maltodextrin (RMD) has been reported to be beneficial. However, there has been no comprehensive quantification of the effect of RMD on the intestinal environment. Therefore, this study aimed to quantify the effects of RMD on the intestine, especially the intestinal microbiome and metabolome profiles. A randomized, double-blind, and controlled trial was conducted in 29 Japanese subjects, whose hemoglobin A1c (HbA1c) levels are larger than 6% (Clinical trial no. UMIN000023970, https://upload.umin.ac.jp/cgi-open-bin/ctr_e/ctr_view.cgi?recptno=R000027589). The subjects consumed RMD or placebo twice per day for 24 weeks. Blood and fecal samples were collected before and after the intake. The intestinal environment was assessed by a metabologenomics approach, involving 16S rRNA gene-based microbiome analysis and mass spectrometry-based metabolome analysis. The intake of RMD increased the levels of *Bifidobacterium* and *Fusicatenibacter* and decreased deoxycholate levels. Additionally, intake of RMD lowered the levels of some opportunistic virulent metabolites, such as imidazole propionate and trimethylamine, in subjects with an initially high amount of those metabolites. RMD may have beneficial effects on the gut environment, such as commensal microbiota modulation and reduction of virulence metabolites, which is known as a causative factor in metabolic disorders. However, the effects of RMD partially depend on the gut environmental baseline.

## Introduction

In recent years, there have been multiple reports that the intestinal microbiota plays a role in the pathogenesis of several diseases, such as colorectal cancer, inflammatory bowel disease, obesity, and diabetes (Qin et al., 2012; Goodrich et al., 2014; Lloyd-Price et al., 2019; Yachida et al., 2019). Additionally, the metabolites produced by the intestinal microbiota also affect human physiological homeostasis. For example, there have been reports on the effects of short-chain fatty acids (SCFAs) (Arpaia et al., 2013; Furusawa et al., 2013; Smith et al., 2013), vitamins (LeBlanc et al., 2013, 2017), secondary bile acids (Yoshimoto et al., 2013), imidazole propionate (ImP) (Koh et al., 2018), and trimethylamine (TMA) (Wang et al., 2011, 2015; Koeth et al., 2013; Tang et al., 2013) on human health and diseases. Prebiotics has emerged as one of the agents that positively impact the intestinal environment. Resistant maltodextrin (RMD), also known as indigestible dextrin, is one of the dietary fibers that have been suggested to have prebiotic functions. The consumption of RMD has been found to increase the total bacterial number, particularly the genus *Bifidobacterium* (Fastinger et al., 2008; Ukhanova et al., 2012; Burns et al., 2018). Additionally, it has been reported that SCFA levels increase upon intake of RMD (Fastinger et al., 2008), but there are no comprehensive analyses of other metabolites and long-term gut microbiota evaluation. In addition, the beneficial effects of RMDs on glucose tolerance have been reported in previous studies (Hashizume et al., 2012). However, the effect of diet, prebiotics, and probiotics on human health varies for each individual and is based on their gut environment (Ferrario et al., 2014; Kato et al., 2014; Kovatcheva-Datchary et al., 2015). Therefore, the purpose of this study was to observe the effects of consumption of RMD on the glucose tolerance and intestinal environment through the following methods: (i) metabologenomic analysis (Ishii et al., 2018) of intestinal microbiota, and metabolites before and after the consumption of RMD; and (ii) a comparative analysis of microbiome and metabolome in individuals that presented with differences after the consumption of RMD.

## Results

### Basic Information on the Subjects and Blood Tests

**FIGURE 1:**
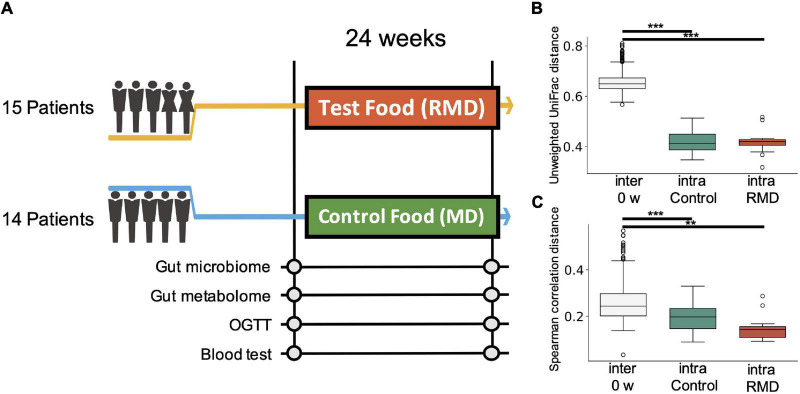
Experimental design of the study and comprehensive analysis of gut microbiome and metabolome profiles. **(A)** Flow diagram of the double-blind, placebo-controlled parallel group study. Fecal and blood samples were collected 0 and 24 weeks after test food (resistant maltodextrin: RMD) or control food (normal maltodextrin: MD) intervention. Gut microbiome and metabolome analyses, oral glucose tolerance test (OGTT), and blood tests were conducted. **(B)** Box plot representing distribution of unweighted UniFrac distance for the gut microbiome profiles among the samples from different subjects at the same time point (inter 0 week) and the distance between the samples from the same subject in the control group and RMD group (**p < 0.005, ***p < 0.0005; the Dunn’s test). **(C)** Box plot representing the distribution of the Spearman’s correlation distance for intestinal metabolome profiles among the samples from different subjects at the same time point (inter 0 week) and the distance between the samples from the same subject in the control group and RMD group (**p < 0.005, ***p < 0.0005; the Dunn’s test).

To evaluate the effect of RMD, a randomized, placebo-controlled and parallel-group trial was performed for 24 weeks (Figure 1A). A total of 30 Japanese participants with high hemoglobin A1c (HbA1c) levels passed the inclusion criteria, and 29 participants completed the intervention period.

To investigate the benefits of RMD, parameters obtained in the clinical blood test were compared with baseline or control group values. For the test group, there was a significant decrease in insulin incremental area under the curve (iAUC) and area under the curve (AUC), as well as an increase in blood glucose iAUC when compared with the baseline after false dicovery rate (FDR) correction (*p* < 0.10). Items with significant differences before FDR correction have been described in Supplementary Table 1.

### Effect of Resistant Maltodextrin on the Intestinal Microbiome and Metabolome Profiles

To evaluate the effect of RMD on the gut microbiota and gut metabolic profile, we conducted fecal microbiome and metabolome profiling by the metabologenomics approach. Beta-diversity analysis showed that the microbiome and metabolome composition of the same subject were similar within the duration of the test food intake or placebo food intake. Comparisons of inter-individual, RMD-intraindividual, and maltodextrin (MD)-intra-individual distances showed significant differences in the microbiome and metabolome profile (Figures 1B,C; the Kruskal-Wallis test, *p*-values were 2.59 × 10^–18^ and 3.99 × 10^–8^ in the microbiome and metabolome data, respectively), suggesting that the individual difference was larger than the influence of RMD or placebo food consumption.

**FIGURE 2:**
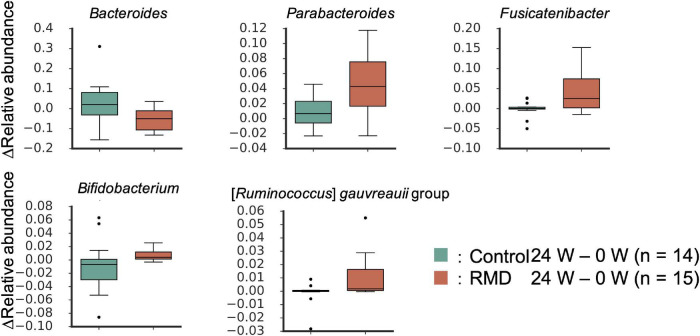
Significantly altered gut microbes following RMD intake. Box plots showing significant differences between the control group and RMD group (*p* < 0.05, the Wilcoxon rank sum test) and between before and after RMD intake (*q* < 0.10, the Wilcoxon signed-rank test).

**FIGURE 3:**
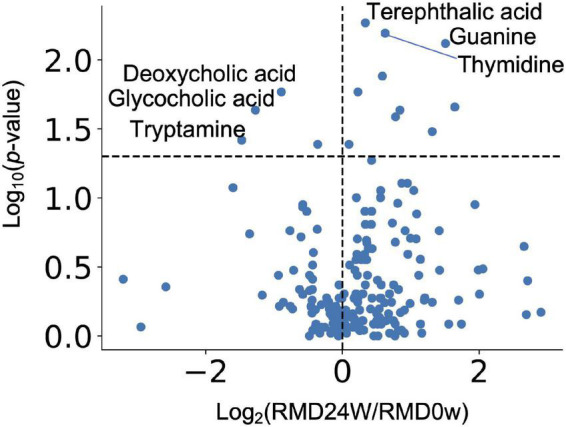
Effect of RMD intake on intestinal metabolite concentrations. Volcano plot representing the influence of the test food on each intestinal metabolite. *X* axis indicates the magnitude of influence (log fold change of mean value of corresponding metabolite abundance after 24 weeks of intervention relative to the control time point). *Y* axis indicates the significance of influence (logarithmic value of *p*-value). A dotted line has been drawn at −log_10_*p*-value (*p* = 0.05).

Next, we performed two types of tests to evaluate the relative abundance of each microbiome and the relative area of each metabolite. In the case of the microbiome, the comparison between individuals (intra-group) within the placebo group (see “Bioinformatics and Statistical Analysis” section in Methods) showed significant differences for some bacteria. However, there was no significant difference in the relative abundance of bacteria after FDR correction. We focused on the detected bacteria with significant differences in abundance when compared with that at the baseline. The *Bacteroides* and four genera, such as *Parabacteroides*, *Fusicatenibacter*, *Bifidobacterium*, and [*Ruminococcus*] *gauvreauii* groups, were significantly decreased and increased after FDR correction, respectively (Figure 2 and Supplementary Table 1). Metabolome analysis showed no significant difference in the metabolites after FDR correction in both the tests. The significant differences before FDR correction in both the tests have been listed in Supplementary Table 1. Noteworthy, the levels of deoxycholic acid and glycocholic acid were significantly decreased compared with that at the baseline (Figure 3).

### Resistant Maltodextrin Intake Alters the Concentration of Each Intestinal Metabolite Depending on Their Baseline Concentrations

**FIGURE 4:**
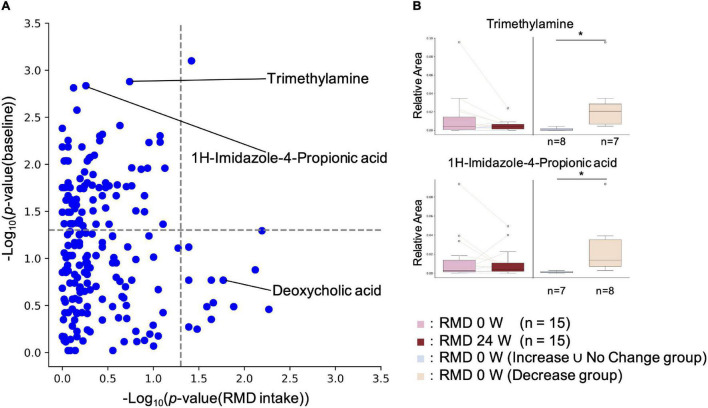
Effect of RMD intake on intestinal metabolite concentrations depends on the individual’s intestinal metabolite concentration at the baseline. **(A)** *X* axis indicates the *p*-value of the Wilcoxon signed-rank test for each metabolite between RMD 0 week and RMD 24 weeks groups. *Y* axis indicates the *P*-value of the Wilcoxon rank sum test for each metabolite between the two groups, increased and decreased groups after RMD intake, at the baseline. If the subjects with increased amounts of each metabolite were more than the subjects with decreased amounts of each metabolite after RMD intake, then Increase group and Decrease ∪ No Change group were compared by the Mann-Whitney *U* test; if the subjects with decreased amounts of each metabolite were more than the subjects with increased amounts of each metabolite, then Decrease group and Increase ∪ No Change group were compared by the Wilcoxon rank sum test. **(B)** Two representative metabolites are shown by boxplots. The left two boxes show the amount of the metabolite in RMD 0 week (left) and RMD 24 weeks (right) (*p < 0.05; the Wilcoxon signed-rank test), and the right two boxes show the amount of the metabolites at the baseline in RMD not decrease group, and RMD decrease group (*p < 0.05; the Mann-Whitney *U* test).

It has been reported that the effects of diet, prebiotics, and probiotics on human health vary depending on the intestinal environment of an individual (Ferrario et al., 2014; Kato et al., 2014; Kovatcheva-Datchary et al., 2015). Therefore, we comprehensively analyzed the intestinal metabolites, whose resulting variability depended on their baseline concentrations. For each metabolite, statistical tests were performed on the baseline values for the increased and decreased groups. If a significant difference was detected, it was assumed that the increase or decrease depended on the baseline value. The results showed significant differences for 83 metabolites, indicating that the effect of RMD intake on the concentration of each intestinal metabolite depends on the concentration of the metabolite at the baseline (Figure 4 and Supplementary Table 2).

### Intestinal Environmental Features of Subjects With Reduced Concentrations of Opportunistic Virulent Metabolites After Resistant Maltodextrin Intake

The gut microbiota metabolizes histidine to Imidazole propionate (ImP), which has been found to contribute to the pathophysiology of type 2 diabetes (Koh et al., 2018). Our metabolome analysis showed that the ImP levels after RMD intake depended on the initial amount of ImP before RMD ingestion. To investigate the reason for the reduction of intestinal ImP, we analyzed the gut microbiome and metabolome by metabologenomics approach based on the following two criteria: (1) there was a significant difference in the relative abundance of the gut microbiota and the relative amount of the metabolites during comparison of the subject with reduced ImP after RMD intake with the rest of the subjects; and (2) there was a significant difference between before and after RMD intake only in the subjects with decreased ImP after RMD intake. There were no significant differences in the relative abundance of the gut microbiota in both analyses. However, in subjects with reduced ImP, several metabolites, such as TMA, were more prevalent at baseline, and TMA was significantly reduced as well (Supplementary Table 3). TMA is produced from dietary choline or carnitine by the gut microbiota and is metabolized to trimethylamine N-oxide (TMAO) in the liver. TMAO has been reported to increase the risk of arteriosclerosis (Wang et al., 2011; Tang et al., 2013). After a similar analysis for TMA, we found that intestinal microbes, such as *Desulfovibrio*, and metabolites, such as choline and TMA, could be used as biomarkers for TMA reduction after RMD intake (Supplementary Table 4).

### Correlation Analysis Between Gut Microbes and Opportunistic Virulent Metabolites

We found that the two opportunistic virulent metabolites ImP and TMA were reduced in the subjects with a high amount of the two metabolites at the baseline. To explore the gut microbes responsible for the reduction in the levels of these two metabolites, we performed correlation analyses between each microbial genus and ImP or TMA (Supplementary Tables 5, 6). In the case of ImP, 7 genera (*Escherichia-Shigella*, *Allisonella*, [*Ruminococcus*] *gnavus* group, *Tyzzerella* 4, uncultured *Lachnospiraceae*, *Collinsella*, *Desulfovibrio*; Spearman’s *r* > 0.36, *p* < 0.05) had positive correlations, and 10 genera (*Ruminococcaceae* UCG-013, *Phascolarctobacterium, Anaerostipes, Faecalibacterium, Ruminococcus* 1, *Ruminococcaceae* UCG-005, *Lachnospiraceae* NK4A136 group, uncultured *Ruminococcaceae, Christensenellaceae* R-7 group, and *Ruminococcaceae* UCG-002; Spearman *r* < −0.36, *p* < 0.05) had negative correlations. In case of TMA, 8 genera had positive correlations (*Allisonella, Desulfovibrio*, Prevotellaceae NK3B31 group, *Ruminococcus* 2, *Clostridium sensu stricto* 1, *Megasphaera, Escherichia-Shigella*, and uncultured *Lachnospiraceae*; Spearman’s *r* > 0.36; *p* < 0.05), and 3 genera (*Ruminococcaceae* UCG-002, *Christensenellaceae* R-7 group, and *Ruminococcaceae* UCG-005; Spearman’s *r* < −0.36, *p* < 0.05) had negative correlations. Interestingly, 7 genera, *Escherichia-Shigella*, *Allisonella*, uncultured *Lachnospiraceae*, *Desulfovibrio*, *Christensenellaceae* R-7 group, *Ruminococcaceae* UCG-002, and *Ruminococcaceae* UCG-005 were common in the case of both ImP and TMA, suggesting that these genera might have contributed to the reduction of the intestinal ImP and TMA levels by RMD intake. In addition, we noted that a positive correlation was found between ImP and TMA (Spearman’s *r* = 0.634, *p* = 0.000170).

## Discussion

Various indicators can be obtained from the measurement of blood glucose levels—the indicators of blood glucose levels estimated at any point in time to indicators of the blood glucose levels on the same day to those after a few or several months later (e.g., HbA1c, GA, 1,5-AG). Unexpectedly, in our study, HbA1c increased as a result of consumption of both RMD food and the placebo. It has been reported that HbA1c has seasonal patterns (Tseng et al., 2005); thus, the observed increment might have been affected by seasonal variations in this study. In the case of glucose tolerance improvement, insulin AUC and iAUC decreased after the intake of RMD; however, the blood glucose iAUC increased significantly. This result is conflicting, as it cannot explain the traditional relationship between decreasing insulin levels and improvement in glucose tolerance. However, glycoalbumin (GA) and 1,5-anhydroglucitol (1,5-AG) levels did not show significant differences. It is known that if glucose tolerance is impaired, levels of indicators that represent blood glucose conditions, such as GA and 1,5-AG, also deteriorate. These results show the limitation of the present study in terms of elucidation of the effect of RMD on the improvement of glucose tolerance.

Some bacterial genera were significantly altered by the ingestion of RMD. Previous studies have reported that *Bifidobacterium* increase following ingestion of RMD (Burns et al., 2018). *Bifidobacterium* has been reported to produce mainly one SCFA, acetic acid, in the intestinal lumen. We have previously reported that acetic acid produced by bifidobacteria upregulates the barrier function of intestinal epithelial to prevent Enterohemorrhagic *Escherichia coli* O157:H7 infection in a mouse model (Fukuda et al., 2011). Thus, increased numbers of *Bifidobacterium* may also upregulate the intestinal mucosal defense through acetic acid production in the human intestine. *Fusicatenibacter* has been known to degrade the polysaccharide inulin (Takada et al., 2013). Thus, we hypothesized that *Fusicatenibacter* could metabolize RMD as a source of nutrients for their growth in the human intestine. Also, it has been shown that the abundance of *Fusicatenibacter saccharivorans* decreases in patients with active ulcerative colitis, but increases in patients with quiescent ulcerative colitis, and the bacterium suppresses intestinal inflammation through IL-10 secretion (Takeshita et al., 2016). Thus, the increase in the abundance of *Fusicatenibacter* after RMD intake may be valuable for the suppression of intestinal inflammation through IL-10 induction in the human intestine.

Although not significant after FDR correction, glycocholate and deoxycholate levels were reduced after RMD intake when compared to the baseline levels. Deoxycholate has been reported to trigger liver cancer when it reaches the liver after reabsorption from the intestinal tract (Yoshimoto et al., 2013). Furthermore, we previously reported that the fecal concentration of deoxycholate was overrepresented in the early stages of a colorectal cancer patient (Yachida et al., 2019). Therefore, the intake of RMD may reduce the risk of liver and colorectal cancer development. Bile acids promote micelle formation in the gastrointestinal (GI) tract. RMD has been reported to stabilize micelles (Kishimoto et al., 2009) and, thus, may reduce the required amount of bile acid to produce deoxycholate by the gut microbiota in the GI tract.

Subjects with reduced TMA levels after RMD intake had a significantly higher abundance of *Desulfovibrio*, and choline and TMA levels at the baseline. There are three possible pathways for TMA biosynthesis in the intestine, which are: (1) the pathway is undertaken by the dietary TMA in food to reach the intestines (Zhang et al., 1999), (2) the metabolism of dietary phosphatidylcholine/choline into TMA by gut microbiota, and (3) the metabolism of dietary carnitine into TMA by gut microbiota. It has been reported that phosphatidylcholine forms micelles with bile acids. As shown in *in vitro* studies, RMD stabilizes the micelles (Kishimoto et al., 2009); thus, RMD may reduce TMA production by inhibiting phosphatidylcholine hydrolysis. Subjects with high levels of TMA in their feces may exhibit decreased risk of arteriosclerosis through the intake of RMD. Subjects who had decreased ImP levels in their feces after RMD intake had significantly higher initial levels of ImP and TMA. Since ImP and TMA were positively correlated and some bacterial biomarkers for their reduction by RMD intake were common, they might be controlled by similar mechanisms. It has been reported that ImP is produced by the gut microbiota from histidine (Koh et al., 2018). TMA is produced from the metabolism of choline and carnitine, respectively. The metabolic pathways are not close, suggesting that the bacteria producing them may be similar. Therefore, the decrease in TMA may have reduced the TMA-producing bacteria, which in turn has reduced the production of ImP. ImP production may be stimulated by positively correlated bacteria (*Escherichia-Shigella*, *Allisonella*, [*Ruminococcus*] *gnavus* group, *Tyzzerella* 4, uncultured *Lachnospiraceae*, *Collinsella*, and *Desulfovibrio*) and/or inhibited by negatively correlated bacteria (*Ruminococcaceae* UCG-013, *Phascolarctobacterium, Anaerostipes, Faecalibacterium, Ruminococcus* 1, *Ruminococcaceae* UCG-005, *Lachnospiraceae* NK4A136 group, uncultured *Ruminococcaceae, Christensenellaceae* R-7 group, and *Ruminococcaceae* UCG-002). It has been reported that ImP is produced by the gut microbiota from histidine *via* urocanate, and active sites of urocanate reductase (UrdA) must contain tyrosine or methionine (Koh et al., 2018). In the report, it was shown that *Escherichia coli* K-12 produced UrdA, but tyrosine and methionine were absent from their active site. UrdA is not expressed in *Allisonella*, [*Ruminococcus*] *gnavus* group, *Tyzzerella* 4, *Collinsella*, and *Desulfovibrio* (Koh et al., 2018). However, it has been reported that different strains of gut microbiota have different functions, even within the same bacterial species (Tett et al., 2019). Of these bacteria, an unreported strain may produce ImP. Additionally, we observed that ImP decreased in subjects with high levels of ImP at the baseline. As ImP has been reported to impair insulin signaling through mechanistic target of rapamycin complex 1 (Koh et al., 2018), subjects with high levels of ImP in their feces may exhibit improved glucose tolerance through RMD intake.

There are two limitations to this study. First, this study is a parallel-group study. For evaluating the effects of test food among individuals with highly diverse gut microbiota, it is preferred to conduct placebo-controlled crossover tests. Since our evaluations were based on a comparison between MD groups and RMD groups, the effects which were smaller than individual differences may have been overlooked. In addition, this report comprehensively analyzed and compared gut microbiome and metabolome profiles by metabologenomics approach among subjects who showed some effects after consuming RMD as compared to those who did not display any effects. To evaluate the effects of RMD in each individual more accurately, a crossover study in which both RMD and placebo food are fed to each study participant will be necessary. Second, this study is a long-term (24 weeks) study without intermediate sampling. In other words, the data of this study were obtained with a much longer period of intervention than previous studies, the majority of studies were designed with the intervention period of 4–8 weeks. Although we mentioned that the increase of *Bifidobacterium* observed in this study was consistent with previous studies, the comparison may not be appropriate due to the different duration of the intervention period. In addition, dynamics of gut microbiota and metabolome were not observable since no intermediate samples were available to study.

In this study, a randomized control study was performed to quantify the effects of RMD on the intestinal environments using metabologenomics. As a result, we found that the abundance of *Fusicatenibacter*, which suppresses inflammation through IL-10, was also increased. Deoxycholate, which may cause liver and colorectal cancers, was decreased by the intake of RMD. Additionally, it was found that if there were high levels of opportunistic virulent metabolites, such as ImP and TMA before RMD intake, then, these levels decreased upon intake of RMD.

## Methods

### Trial Design and Recruitment

In this study, we conducted a randomized, double-blind, placebo-controlled, and parallel-group clinical trial between September 2016 and February 2017. Another test period was set up as the target sample size was not accomplished in the first recruitment period (Figure 1, Supplementary Figure 1, Supplementary Tables 7–9, and Supplementary Protocol). The baseline clinical characteristics were similar in both groups (Supplementary Table 10). Each subject consumed test food or a placebo for 24 weeks. We used RMD as the test food and normal MD for the placebo. RMD used in this trial was produced by Matsutani Chemical Industry Co., Ltd. (Itami, Japan; trade name: Fibersol-2; Dietary fiber: 90%). The calories in the test food and placebo were standardized in the trial although their weights were different; the test food and placebo weighed 10 and 3 g, respectively. The test food or placebo was dissolved in potable water and was consumed twice a day. The test food and placebo were individually packaged to prevent their recognition. Fecal sampling, oral glucose tolerance test (OGTT), and clinical blood tests were performed for each subject before the start of the study and at the end of the 24 weeks. Clinical blood tests included the measurement of HbA1c, blood glucose level, insulin, GA, 1,5-AG, glucagon-like peptide-1 (GLP-1), and tumor necrosis factor-alpha (TNF-α). For OGTT, the glucose and insulin concentrations were measured before administration and at 30, 60, 90, and 120 min after the administration of 75 g of glucose. The AUC and iAUC were calculated using the trapezoid model. This trial recruited males and females aged 20 years and older with high HbA1c levels (see Supplementary Table 8 for detailed inclusion/exclusion criteria). Preliminary blood tests were conducted on the participants and 30 subjects who fulfilled the age, male-female ratio, and HbA1c level criteria from the preliminary results were selected. In addition, during the study period, subjects were restricted from consuming foods that contained bacteria, such as yogurt, and foods that were explicitly labeled as high in fiber, oligosaccharides. One subject (Subject 10) dropped out of this study as advised by a doctor; 29 subjects completed the main trial.

### Ethics Approval

The human rights of the subjects who participated in this study were protected at all times, and the study observed the Helsinki Declaration and Ethical Guidelines on Epidemiological Research in Japan referring to cases concerning standards for clinical trials of drugs. This randomized controlled trial was conducted with the approval of the clinical trial ethics review committee of the Chiyoda Paramedical Care Clinic (UMIN-CTR, Trial number: UMIN000023970).

### DNA Extraction

The DNA extraction from stool samples was performed according to a previously described method (Murakami et al., 2015). From the extracted DNA samples, the V1-V2 region of the bacterial 16S rRNA gene region was amplified using universal primers 27F-mod and 338R (Kim et al., 2013). The DNA sequencing was performed with the DNA amplicons using the paired-end mode with 600 cycles (MiSeq, Illumina).

### Metabolite Extraction and Analysis

Metabolite extraction from feces was performed according to a previously described method (Kim et al., 2017). Briefly, samples were lyophilized using the VD-800R lyophilizer (TAITEC) for at least 24 h. Freeze-dried feces were disrupted with 3-mm zirconia beads by vigorous shaking (1,500 rpm for 10 min) using the Shake Master Neo (Bio-Medical Science). Internal standards [20 μM each of methionine sulfone and D-camphor-10-sulfonic acid (CSA)] dissolved in 500 μL of methanol were added to 10 mg of the disrupted feces. The samples were further disrupted with 0.1-mm zirconia/silica beads by vigorous shaking (1,500 rpm for 5 min) using the Shake Master Neo. Next, 200 μL of ultrapure water and 500 μL of chloroform were added before centrifugation at 4,600 × *g* for 15 min at 20°C. Subsequently, 150 μL of the supernatant was transferred to a centrifugal filter tube (UltrafreeMC-PLHCC for Metabolome Analysis, Human Metabolome Technologies) to remove proteins and lipids. The filtrate was centrifugally concentrated, and the pellet was resuspended in 50 μL of ultrapure water immediately before the capillary electrophoresis time-of-flight mass spectrometry (CE-TOFMS)-based metabolome analysis. The obtained metabolomics data has been shown in Supplementary Tables 11, 12.

### Bioinformatics and Statistical Analysis

The forward and reverse sequencing reads of each sample were merged using the vsearch version 1.9.3 (options: –fastq_maxee 9 –fastq_truncqual 7 –fastq_maxdiffs 300 –fastq_maxmergelen 450 –fastq_minmergelen 250) (Rognes et al., 2016). Primer base nucleotides were removed by cutadapt (options: −O 13 −m 50 −M 450 −q 0; −e option is not used in cut 5′ primer, use 0.3 in cut 3′ primer). The Phix fragments were then removed using the Bowtie2 version 2.1.0 (Langmead and Salzberg, 2012). Bowtie2 option was set to default. Fragments with an average quality of less than 25 were removed by the in-house script. All fragments were mapped to the SILVA SSU Ref NR database version 128 (Quast et al., 2012) using Bowtie2 (Option: –no-HD –no-sq –no-unal −I 280 −X 400 –fr –no-discordant –phred33 −D 15 −R 10 −N 0 −L 22 −i S,1,1.15 −q). From the remaining fragments (26,784 ± 3,474), 10,000 fragments were subsampled and used for analysis.

All statistical analyses were performed using Python (version 3.7.3). The UniFrac distances were used for the estimation of beta diversity. The Wilcoxon-Mann-Whitney test was used to compare the two groups. The false discovery rate was adjusted using the Benjamini-Hochberg false discovery rate correction (FDR-BH) method. For gut microbiome analysis, taxonomic composition data of the genus and operational taxonomic unit (OTU) were used. Of these, OTU-level data was used only for the calculation of the beta diversities. The genus-level data was used for other statistical analyses. For gut metabolome analysis, the relative area of metabolites was used. The following comparisons were performed between the two different sets:

(i) Differences in the 24-week intake and baseline of each subject in the RMD and MD groups.
(ii) 24-weeks of RMD intake and baseline.

Considering the placebo effect, test (i) was required. However, the effect of RMD on the intestinal bacteria and metabolites was smaller than that of the individual differences. Therefore, the effect of food may not have been detected by test (i). Test (ii) was, thus, performed to increase the statistical significance of the test. As for the microbiome data, only genera with an average relative abundance of 0.001 or more were used.

### Definition of Baseline-Dependent Metabolites

For each metabolite, subjects were divided into Increase (fold change > 1), Decrease (fold change < 1), and No Change groups (fold change = 1) by comparing results obtained before and after intake. For the initial value, (1) RMD increase group and Decrease ∪ No Change group and (2) RMD decrease group and Increase ∪ No Change group was compared by the Mann-Whitney *U* test. Items with a *q* value after FDR-BH correction of less than 0.10 were extracted. Since the α-error of the Mann-Whitney *U* test becomes high if the proportions of the groups are different, the test was not performed if the number of people in a group was less than 5. Additionally, when a metabolite is not detected from the majority of the samples, its behavior is difficult to identify. Therefore, if a metabolite was not detected from more than half of the samples, it was excluded from this analysis.

## Data Availability Statement

The obtained 16S rRNA gene sequence data are available in the DDBJ DRA (DRA accession number: DRA010060). The obtained blood test data has been shown in Supplementary Table 13.

## Ethics Statement

The studies involving human participants were reviewed and approved by Committee of the Chiyoda Paramedical Care Clinic. The patients/participants provided their written informed consent to participate in this study.

## Author Contributions

YoM, SM, YK, TY, and SF planned the study. YuM and MI contributed to the analysis of the primary sequence data of fecal samples. YN contributed to the statistical analysis of the data and wrote the first draft. YN, YoM, SM, YK, TY, and SF contributed to the completion of the manuscript. All authors read and approved the final manuscript.

## Conflict of Interest

YN, YuM, and MI are employees of Metagen, Inc. YoM, TY, and SF are founders of Metagen, Inc. SM and YK are employees of Matsutani Chemical Industry Co., Ltd. The authors declare that this study received funding from Metagen, Inc. or Matsutani Chemical Industry Co. Ltd. The funders had the following involvement in the study: Metagen, Inc., was involved in study design, data collection, statistical analysis, and preparation of the manuscript. Matsutani Chemical Industry Co., Ltd., was involved in study design and review of the manuscript.

## Publisher’s Note

All claims expressed in this article are solely those of the authors and do not necessarily represent those of their affiliated organizations, or those of the publisher, the editors and the reviewers. Any product that may be evaluated in this article, or claim that may be made by its manufacturer, is not guaranteed or endorsed by the publisher.
