# Supplementary figures and images for: Resistant Maltodextrin Intake Reduces Virulent Metabolites in the Gut Environment: A Randomized Control Study in a Japanese Cohort
Source: Front Microbiol. 2022 May 4;13:644146. doi: 10.3389/fmicb.2022.644146 (PMC9116438; doi:10.3389/fmicb.2022.644146)

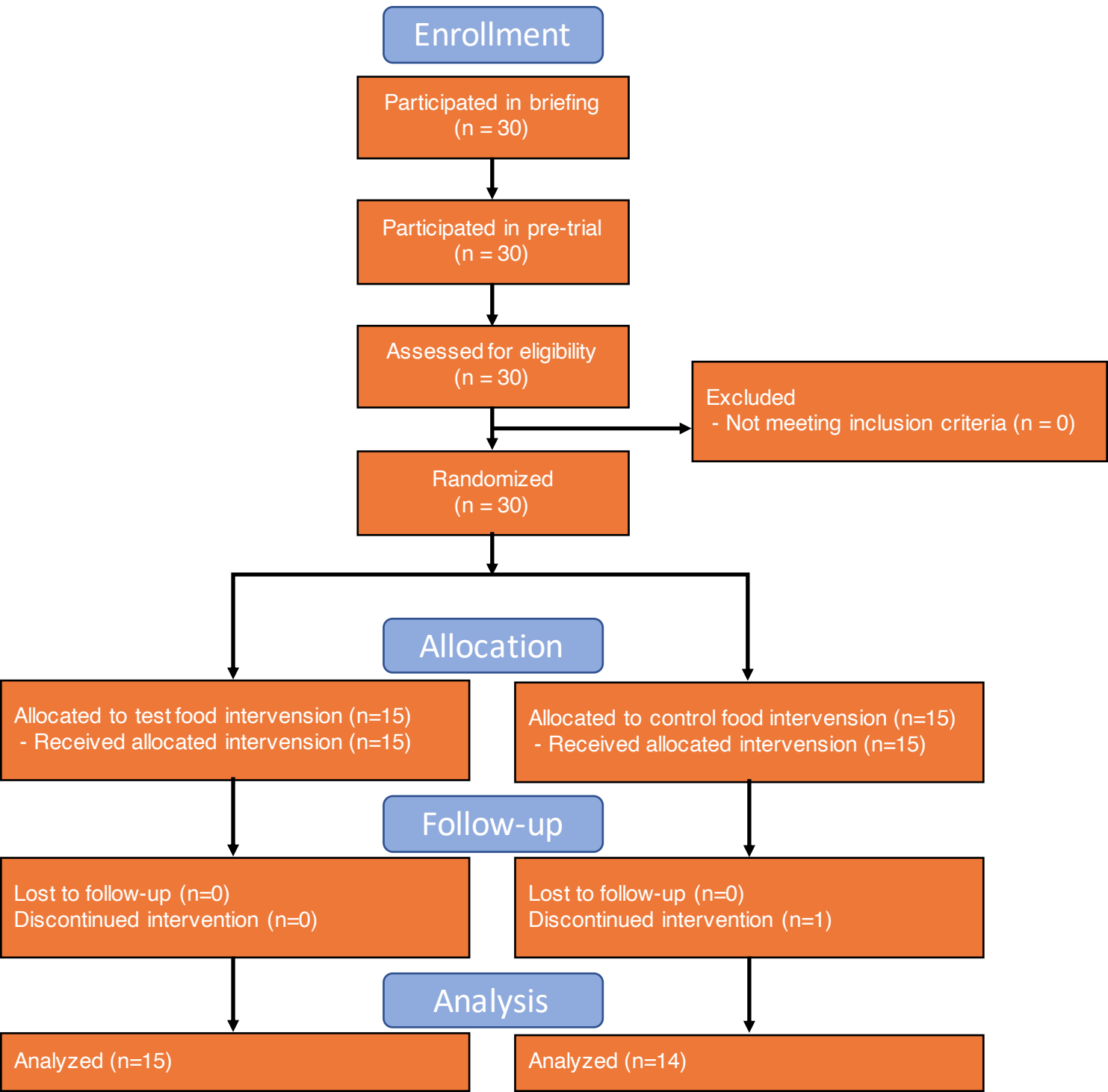

Supplement: Supplementary file 2 [file Image_1.pdf]
